# Do athletes require a higher tidal volume? An approach using predicted versus measured PFTs

**DOI:** 10.1186/cc9619

**Published:** 2011-03-11

**Authors:** P Myrianthefs, G Baltopoulos

**Affiliations:** 1 School of Nursing, Athens University, 'Agioi Anargyroi' Hospital, Kifissia, Greece

## Introduction

Tidal volume (Vt) for ALI/ARDS is 6 ml/kg. However, professional athletes have higher forced vital capacity (FVC) and forced expiratory volume in 1 second (FEV1) than predicted for the same body weight and thus a higher Vt could be required.

## Methods

To answer this question, the predicted Vt (Vt Pr = 6 ml/kg) was calculated as the percentage of measured (Ms) and predicted (Pr) FEV1 and FVC, and their difference (Δδ = Ms - Pr) was extracted to calculate the additional Vt (VtA) required according to measured PFTs. Values are expressed as the mean (SEM).

## Results

We included 156 males and 95 females of mean duration of sporting of 11.8 (6.4) and 11.6 (6.9) years, respectively. Ms and Pr FEV1 and FVC were recorded (data not shown). Vt Pr, the percentage to Ms and Pr FEV1 and FVC, their difference Δδ, the corresponding VtA and the new Vt (VtN) are presented in Table 1.

**Table 1:** Calculations to extract additional Vt according to predicted and measured PFTs

		Vt to PFTs (%)	Vt to PFTs (%)	δ	VtA (ml)	VtN (ml)
	**Vt Pr (ml)**	**FEV1 Pr**	**FEV1 Ms**	**FEV1**	**FEV1**	**FEV1**

Males	476.3 (6.1)	10.0 (016)	10.9 (0.13)	0.87 (0.1)	47.7 (5.1)	524 (8.1)
Females	384.9 (6.0)	10.4 (0.2)	11.1 (0.16)	0.67 (0.1)	29.9 (4.7)	414.8 (7.8)

	**Vt Pr (ml)**	**FVC Pr**	**FVC Ms**	**FVC**	**FVC**	**FVC**

Males	476.3 (6.1)	8.35 (0.12)	9.1 (0.1)	0.75 (0.1)	51.3 (5.7)	527 (9.1)
Females	384.9 (6.0)	8.9 (0.17)	9.6 (0.14)	0.67 (0.1)	34.7 (5.5)	419 (8.6)

## Conclusions

According to our hypothesis an additional Vt of 0.6 for males and 0.5 ml/kg for females maybe required for professional athletes under mechanical ventilation.

